# Mucormycosis, Diabetes and COVID-19 Pneumonia: Unleashing the Facts

**DOI:** 10.7759/cureus.29555

**Published:** 2022-09-25

**Authors:** Minal Shastri, Darshankumar M Raval, Dirgha Patel, Aastha B Patel, Aakriti Chopra, Vaishnavi M Rathod, Riya Dobariya, Nilay S Patel, Nupur H Patel, Apurva Patel, Devang M Gohel

**Affiliations:** 1 Department of General Medicine, Sir Sayajirao General (SSG) Hospital & Medical College, Baroda, Vadodara, IND

**Keywords:** covid-19 retro, amphotericin-b, posaconazole, diabetes, mucormycosis, covid-19

## Abstract

Background

Mucormycosis (MM) is an angioinvasive locally destructive fungal infection. Before the coronavirus disease 2019 (COVID-19) pandemic, it was associated with diabetes (particularly diabetic ketoacidosis), immunosuppressive drugs and trauma. Among its various forms, cerebral invasion is considered to be highly fatal even if with long-term treatment. Treatment with injection amphotericin B (Amph-B) with early surgical interventions is highly efficacious. Liposomal preparation is considered to be superior in the context of fewer side effects.

Methods

We present a single-centre prospective study of 124 patients with MM in a tertiary care hospital. After the approval from the ethics committee, basic information was taken from all patients including all available past history about the COVID-19 infection and treatment. The studied outcomes were discharge, death and number of days of hospitalisation. Secondary objectives were to estimate the association of MM with known risk factors, to find the association of an outcome with various inflammatory markers, to determine adverse events with the use of injection Amph-B and posaconazole and to find the case fatality rate of MM.

Results

In our study, we observed that the number of patients with MM was double in the less than 60 years age group. However, mortality was 33.3% in the elderly as compared to 15.29% in patients less than 60 years of age. The majority of the patients (69.35%) were males, but no significant difference in mortality was seen between males and females. The case fatality rate was 20.97%. Ocular symptoms such as orbital swelling and pain were the common presenting symptoms. Almost all patients (93.54%) were diabetics. The non-diabetic group consisted of only 8 (6.4%) patients, and therefore, the comparison was not possible. A total of 20 (16%) out of 124 patients who had received high-dose steroids showed higher mortality (55%). Maximum patients (65.32%) had presented with MM following a past COVID-19 infection. However, a significant number of MM patients (20.96%) had a recent COVID infection and had higher mortality (57.69%) compared to their counterparts. The most common site of involvement in our study was the paranasal sinus (50%) and the outcome was the best in those patients whose disease was localised only to the sinuses, although among 14 (11.29%) patients with cerebral involvement, mortality was maximum (42.85%). Renal impairment and dyselectrolytemia were the most common adverse effects of Amph-B, and 46.42% of patients required surgical removal of the local part.

Conclusion

We saw that diabetes was a major contributory factor in the etiopathogenesis of MM. COVID-19 could also be a major causative factor by impairing the immune system; however, further studies at the molecular level are required to establish an association. The use of steroid cannot be the only independent risk factor, and other associated factors must be present. Treatment with antifungal and early surgical intervention had good outcomes. Treatment with conventional lyophilized Amph-B was equally efficacious as lipid-based solutions, but with more side effects. Hypokalemia and hypocalcemia were the most common electrolyte abnormalities associated with the use of injection Amph-B. Uncontrolled diabetes, the severity of the COVID-19 infection at presentation, acidosis, a high C-reactive protein level (above 100) and local brain involvement were associated with a poor outcome.

## Introduction

Mucormycosis (MM) is a rising angioinvasive disease in the backdrop of the coronavirus disease 2019 (COVID-19) infection caused by the ubiquitous filamentous fungi of order Mucorales. It is the third most common persistent mycosis [1]. MM is a rare but hostile opportunistic fungal infection that affects immunocompromised patients (diabetics, patients with ketoacidosis) [2]. Before this pandemic, epidemiological data on mycosis was limited. MM can be classified based on anatomic localization: localized sinus, localized cerebral, sinoorbital, sinocerebral, generalized rhinoorbitocerebral, pulmonary, cutaneous, renal, and disseminated [3]. Initially, the clinical symptoms of MM are similar to other common infections; however, if it is not diagnosed in the early hours, it proves fatal for the patient [4]. The infection typically starts in the sinuses or the lungs following inhalation of the fungal spores. After the multiplication of fungus, the fungus attacks the tissue and blood vessels causing haemorrhage, thrombosis, and necrosis with spread rarely via the bloodstream to various other organs, e.g., the heart, kidney, brain, and gastrointestinal tract. In sinoorbital MM, the fungus swiftly invades through the eye or sinuses directly into the brain within several days [5]. The cutaneous form occurs following trauma [6]. The treatment of choice is amphotericin B (Amph-B), but new azoles, such as posaconazole and isavuconazole, are other useful agents [7]. To our knowledge, not many large-scale studies on MM are available in the literature; here, we present a single-centre study of 124 patients with MM.

## Materials and methods

After getting permission from the Institutional Ethics Committee for Human Research - PG Research (IECHR-PGR), this study was carried out at the Sir Sayajirao General (SSG) Hospital, which is a tertiary care hospital in Vadodara, Gujarat, during May to August 2021. A total of 124 patients fulfilling the inclusion criteria were enrolled. As per the study protocol, the decision to give steroids, remdesivir, and other care was left to the treating physician in COVID-19 patients. Our institute followed standard treatment guidelines given by the Department of Otolaryngology, SSG Hospital, which were made with the help of guidelines released by the Government of Gujarat (which were based on guidelines of the Government of India).

We collected biodata, presenting complaints, past history of steroid exposure within the last six months, past history of diabetes, other known risk factors, like any immunocompromised states, use of mask, on-admission vitals examination in the form of temperature, pulse, blood pressure, random blood glucose, urine ketones, oxygen saturation, on-admission chest X-ray, electrocardiogram (ECG) and arterial blood gas analysis (ABGA). Real-time reverse transcription-polymerase chain reaction (RTPCR) test for COVID-19 was mandatory before admission for any suspected MM patients irrespective of previous RTPCR report status. For patients who were positive (rapid antigen test or RTPCR) for COVID-19 in the past one month and also on admission, repeat RTPCR was recommended every third day till it came negative. Repeat RTPCR was recommended every seventh day till it came out negative in a freshly diagnosed case of COVID-19 infection for someone who never had COVID in the past. P-values were calculated using the chi-square test (www.socscistatistics.com).

MM patients were categorized into three groups according to COVID status: (1) COVID with MM, patients who were COVID rapid antigen test or RTPCR positive on admission irrespective of the past COVID infection status; (2) past COVID, patients who were COVID rapid antigen test or RTPCR positive in the past six months or who had clinical symptoms related to COVID-19, such as fever, cough, breathlessness, loss of taste and smell, diarrhea in past six months, or who underwent an imaging modality procedure (e.g., high-resolution computed tomography, or HRCT, of the chest) suggestive of an atypical viral infection in the past six months; (3) no COVID, patients who were RTPCR negative for COVID on admission and who had neither any above-mentioned COVID-related symptoms nor any imaging modality procedure done, suggestive of an atypical viral infection.

We classified the severity of the COVID-19 infection as follows: (1) mild form, patients who were home quarantined and didn’t require any form of oxygen therapy; (2) moderate, patients who required nasal oxygen therapy or non-rebreather mask (NRBM) support; (3) severe, patients who required noninvasive ventilation, like high-flow oxygen therapy (HFNO), bilevel positive airway pressure (BIPAP) or invasive ventilation.

Patients who were treated with steroids in the past were classified as follows: (1) no steroid, patients who never received steroids in the last six months and (2) patients who received steroids in the last six months. Patients who received steroids were further divided on the basis of the dose of steroids as follows: high dose included injection methylprednisolone (MPSS) ≥80 mg twice a day or injection dexamethasone >8 mg thrice a day or equivalent dose of other steroids. Low dose included injection MPSS <80 mg twice a day or injection dexamethasone ≤8 mg thrice a day or equivalent dose of other steroids.

As per institutional protocol in COVID-positive patients with MM, injection Amph-B was started based on clinical suspicion as imaging and microbiological confirmation were not immediately possible.

**Figure 1:**
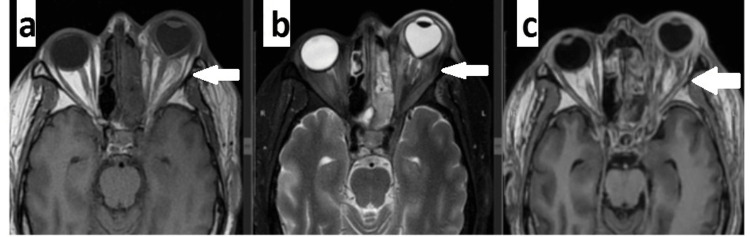
MRI T1W, T2W and T1W post-contrast images showing intraorbital extensions of the MM to the left eyeball (a) The MRI T1W image shows intraorbital extention and proptosis of left eyeball with distortion of the shape of the globe like the "shape of a guitar pick". (b) The MRI T2W image shows abnormal enhancement along the coats of the eyeball with posterior coning of the globe. (c) The MRI T1W post-contrast image shows extensive fat stranding at intra- and extraconal fat planes with muscle edema. All images are suggestive of signs of extensive orbital disease with an increase in intraorbital pressure. MM, mucormycosis

**Figure 2:**
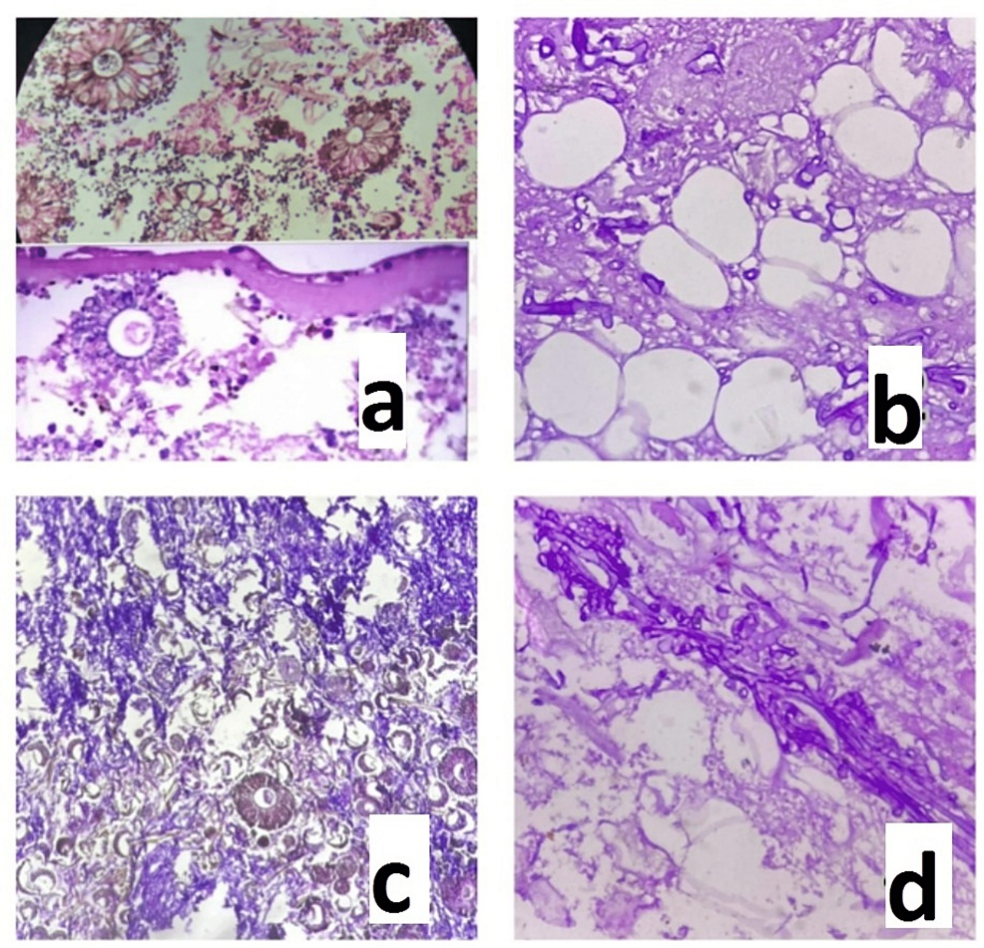
Histopathological examination of the debrided tissue showing mucormycosis (a) Many fruiting bodies of Aspergillus (cleistothecium) along with broad aseptate fungal hyphae of Mucor are seen. Abundant inflammatory cells and few areas of necrosis are also seen in the background. (b) Many broad aseptate fungal hyphae of Mucor along with extensive necrosis in the background are seen. (c) Many broad aseptate fungal hyphae of Mucor along with brown-coloured pigmented hyphae are seen. Many fruiting bodies of Aspergillus (cleistothecium) and sporangium of Mucor are seen. The diagnosis was mixed fungal infection. (d) Many broad aseptate fungal hyphae of Mucor along with extensive necrosis in the background were seen. Hematoxylin and eosin (H&E) stain 40x

In the subgroups of patients who had previously tested positive for COVID and had never had a COVID infection, radiological tests such as paranasal sinus CT (CT-PNS) were performed. Magnetic resonance imaging (MRI) of the orbit plus PNS and brain MRI were done according to the advice of the treating physician and ophthalmologist (Figure 1). Potassium hydroxide (KOH) wet mount and culture of the debrided tissue during functional endoscopic sinus surgery (FESS) and histopathological examination of the specimen removed during debridement were done (Figure 2). We divided MM patients into various subtypes with the help of clinical symptoms and imaging modality: localised sinus, localised cerebral, sinoorbital, sinocerebral, generalised rhinoorbitocerebral, pulmonary, cutaneous, renal, disseminated (sinoorbital + pulmonary, sinocerebral + pulmonary, generalised rhinoorbitocerebral + pulmonary, localised sinus + pulmonary, sinoorbital + cutaneous, localised sinus + cutaneous). Those in whom confirmation was not possible because patients died within two days of COVID with MM due to persistent RTPCR positivity are mentioned separately.

The decision to go for sinoscopy and debridement and other radical surgery was left to treating otolaryngologists, and the decision for retrobulbar Amph-B injection, enucleation, evisceration was left to the ophthalmologist. According to the institutional protocol, all patients who had normal renal function were treated with injection Amph-B lyophilized 1 mg/kg of bodyweight only. Injection Amph-B liposomal 5 mg/kg of bodyweight was reserved for patients who had an estimated glomerular filtration rate (eGFR) less than 10. In patients who had eGFR less than 40, a reduction in the dose of Amph-B lyophilized to 0.5 mg/kg of body weight was recommended. The minimum duration of injection Amph-B was 14 days, but an extended period of injection Amph-B was recommended to be given in patients with extensive necrotic tissue in sinoscopy or if there was no clinical improvement in the form of symptom relief. We have classified renal impairment as an increase in urea or creatinine from baseline and cardiac side effects as any nonspecific chest pain after giving injection Amph-B that may or may not be associated with ECG changes (QT interval prolongation, etc.). The decision to discharge the patient was left to the otolaryngologists after sinoscopy. After discharge, posaconazole was prescribed for 30 days. We followed up with all the patients telephonically after discharge for symptom recurrence or toxicity of drugs prescribed at the end of 15 days.

## Results

Association of age with the outcome in MM

In patients who were more than 60 years of age, 13 out of 39 patients died (33.33%), compared to 13 deaths from a total of 85 younger patients (15.29%); therefore, mortality was double in the elderly population as compared to younger patients (Table 1).

**Table 1:** Association of age with the outcome in mucormycosis The chi-square statistic is 5.24. The p-value is 0.021. The result is significant at p < 0.05.

Age (years)	Discharge	Death	Total
<60	72 (58.07%)	13 (10.48%)	85 (68.55%)
>60	26 (20.97%)	13 (10.48%)	39 (31.45%)
Total	98 (79.04%)	26 (20.96%)	124

Gender

In our study, there were more than double the number of males as compared to females as is shown in Table 2; however, the mortality rates were similar in both the groups (28.35% in males and 22.58% in females).

**Table 2:** Association of gender with the outcome in mucormycosis The chi-square statistic is 0.2144. The p-value is 0.6433. The result is not significant at p < 0.05.

Gender	Discharge	Death	Total
Male	67 (54.03%)	19 (15.32%)	86 (69.35%)
Female	31 (25%)	7 (5.65%)	38 (30.65%)
Total	98 (79.03%)	26 (20.97%)	124

Average time

In our study, the average time from admission to discharge was 24.59 days and the average time from admission to death was 11.5 days.

Presenting complaints

We observed that majority of the patients presented with symptoms related to local site involvement (diplopia 8%, blurring of vision 8.87%, orbital swelling 41.93%, pain 18.54%, ptosis 10.48%, proptosis 1.27%, facial pain 29%, facial numbness 9.67%, deviation of angle of mouth 0.8%, toothache 27.42%, loosening of teeth 9.67%, oral ulcer 4.83%, jaw pain 8.87%, dysphagia 1.27%, nasal bleed 8.87%, hemiparesis 0.8%, decreased urine output 0.8%, skin blackening around eye 1.27% and on thigh 1.27%). Constitutional symptoms were present in only a few patients (fever 11.29%, dry cough 8.87%, sore throat 3.22%, difficulty in breathing 10.48%, abdominal pain 2.42%, generalized weakness 4.83%, headache 25%).

Diabetes and MM

The majority of patients with MM had diabetes as an additional risk factor. Except for one case, all other patients who expired were diabetics (Table 3). Seven out of a total of 124 had diabetic ketoacidosis and all of them recovered. Among 116 patients with diabetes, 61 (52.58%) patients had freshly diagnosed diabetes on admission; 100 (80.65%) out of total 124 patients had a history of unhygienic use of mask in the form of reusing the same mask multiple times without sterilizing it properly; to add to this, all these patients were diabetics.

**Table 3:** Association of diabetes with the outcome in mucormycosis The chi-square statistic is 0.37. The p-value is 0.5429. The result is not significant at p < 0.05.

Diabetes	Death	Discharge	Total
Yes	25 (20.16%)	91 (73.38%)	116 (93.54%)
No	1 (0.81%)	7 (5.65%)	8 (6.46%)
Total	26 (20.97%)	98 (79.03%)	124

Steroids

More than half of patients with MM had never received steroids for any illness (Table 4).

**Table 4:** Association of steroid administration in the past months with the outcome of mucormycosis The chi-square statistic is 17.32. The p-value is 0.00017. The result is significant at p < 0.05.

Steroid use in the past		Death	Discharge	Total
No		11 (8.87%)	54 (43.55%)	65 (52.42%)
Yes	Low dose	4 (3.23%)	35 (28.23%)	39 (31.46%)
	High dose	11 (8.87%)	9 (7.25%)	20 (16.12%)
Total		26 (20.97%)	98 (79.03%)	124

Past treatment

There were 42.74% patients who received anticoagulants and remdesivir, 41.12% received ivermectin and 40.32% received doxycycline as part of COVID-19 treatment in the past.

COVID-19

A significant 13.72% of patients with MM did not have symptoms suggestive of COVID in the past six months (Table 5). We believe that this is an important observation pointing to probably an asymptomatic COVID infection as a risk factor for MM infection. Mortality was the highest (15 deaths out of total 26 patients, 57.69%) in COVID with MM compared to other subgroups (Table 6). Time to death in patients with a severe COVID infection was 3.37 days in contrast to that in patients with MM in a mild COVID infection, which was 22.1 days.

**Table 5:** Association of the severity of COVID-19 with the outcomes of MM MM, mucormycosis; COVID-19, coronavirus disease 2019 ^a^The chi-square statistic is 0.6305. The p-value is 0.7296. The result is not significant at p < 0.05. ^b^The chi-square statistic is 4.38. The p-value is 0.1114. The result is not significant at p < 0.05.

COVID-19 type		Death	Discharge	Total
Past^a^	Mild	5 (4.03%)	44 (35.48%)	49 (39.51%)
	Moderate	4 (3.23%)	23 (18.55%)	27 (21.78%)
	Severe	1 (0.81%)	4 (3.23%)	5 (4.04%)
Total		10 (8.07%)	71 (57.26%)	81 (65.33%)
COVID-19 infection with MM^b^	Mild	4 (3.23%)	6 (4.82%)	10 (8.05%)
	Moderate	7 (5.65%)	1 (0.81%)	8 (6.46%)
	Severe	4 (3.23%)	4 (3.23%)	8 (6.46%)
Total		15 (12.11%)	11 (8.86%)	26 (20.97%)
No COVID infection		1 (0.81%)	16 (12.90%)	17 (13.71%)

**Table 6:** Association of COVID-19 with the outcomes of mucormycosis COVID-19, coronavirus disease 2019 The chi-square statistic is 27.129. The p-value is 0.00001. The result is significant at p < 0.05.

	Death	Discharge	Total
Past COVID-19 infection	10 (8.06%)	71 (57.26%)	81 (65.32%)
COVID-19 with MM	15 (12.10%)	11 (8.87%)	26 (20.97%)
No COVID-19 infection	1 (0.81%)	16 (12.90%)	17 (13.71%)
Total	26 (20.97%)	98 (79.03%)	124

Blood parameters

We observed that a significant number of patients had a normal total count, platelet count, neutrophil-lymphocyte ratio (NLR), and normal lactate dehydrogenase (LDH) and D-dimer levels. A significant 21.78% of the patients had a normal ferritin level (Table 7).

**Table 7:** Values of blood parameters in mucormycosis patients Each cell value is for the number of patients out of total 124 patients. The total of each row is 124 patients. NLR, neutrophil-lymphocyte ratio; LDH, lactate dehydrogenase; ESR, erythrocyte sedimentation rate

Parameter	Normal values	High	Low	Normal
Total count	4500-1000 cells/mcL	48 (38.72%)	3 (2.41%)	73 (58.87%)
Platelet count	150000-450000 per cumm	6 (4.83%)	20 (16.13%)	98 (79.04%)
Total bilirubin	Up to 1.2 mg/dL	1 (0.8%)	1 (0.8%)	122 (98.4%)
Albumin	3.4-5.4 g/dL	20 (16.13%)	84 (67.74%)	20 (16.13%)
NLR	0.78-3.53	74 (59.68%)	1 (0.8%)	49 (39.52%)
LDH	105-333 IU/L	70 (56.45%)	8 (6.45%)	46 (37.1%)
ESR	0-22 mm/hour for men, 0-29 mm/hour for women	95 (76.62%)	-	29 (23.38%)
D-dimer	<250 ng/mL or <0.4 μ/mL	85 (68.54%)	-	39 (31.46%)
Ferritin	24-336 micrograms per liter	97 (78.22%)	-	27 (21.78%)

We observed that among the patients who expired, a significantly large (17 out of 26 expired patients, 65.38%) number of patients had a very high C-reactive protein (CRP) level (Table 8).

**Table 8:** Association of CRP levels with the outcome in mucormycosis CRP, C-reactive protein

CRP level (mg/L)	Death	Discharge		Total
<6	0	6 (4.84%)		6 (4.84%)
6-100	9 (7.26%)	84 (67.74%)		93 (75%)
>100	17 (13.71%)	8 (6.45%)		25 (20.16%)
Total	26 (20.97%)		98 (79.03%)	124

Arterial blood gas analysis on admission

As is clear from Table 9, acidosis was a risk factor for death in MM as all patients with acidosis expired (100% mortality). However, the patient subgroup size in this category in our study was only 9.

**Table 9:** Association of arterial blood gas analysis (done on admission) with the outcome in mucormycosis

Arterial blood gas analysis	Death	Discharge	Total
Acidosis (respiratory or metabolic)	9 (7.26%)	0	9 (7.26%)
Alkalosis (respiratory or metabolic)	5 (4.03%)	18 (14.52%)	23 (18.55%)
Normal	12 (9.68%)	80 (64.51%)	92 (74.19%)
Total	26 (20.97%)	98 (79.03%)	124

Chest X-ray on admission

The outcome was the best in patients with no or mild radiological involvement of the lungs in contrast to patients having the involvement of more than two zones wherein the mortality was 44.11% (15 expired out of 34 patients with more than two lung zone involvements) (Table 10).

**Table 10:** Association of lung involvement with the outcome in mucormycosis The chi-square statistic is 18.7335. The p-value is 0.000086. The result is significant at p < 0.05.

Chest X-ray	Death	Discharge	Total
≤2 zone	6 (4.84%)	17 (13.71%)	23 (18.55%)
>2 zone	15 (12.10%)	19 (15.32%)	34 (27.42%)
Normal	5 (4.03%)	62 (50%)	67 (54.03%)
Total	26 (20.97%)	98 (79.03%)	124

Type of MM and its association with discharge and death

Despite cerebral involvement, 57.14% of patients (8 out of total 14 patients) in our study were successfully treated and discharged (Tables 11, 12).

**Table 11:** Association of the type of MM with the outcome MM, mucormycosis; COVID-19, coronavirus disease 2019; RTPCR, reverse transcription-polymerase chain reaction

Type of MM		Death	Discharge	Total
Localised sinus		1 (0.81%)	61 (49.19%)	62 (50%)
Localised cerebral		0	0	0
Sinoorbital		4 (3.23%)	23 (18.53%)	27 (21.76%)
Sinocerebral		4 (3.23%)	3 (2.42%)	7 (5.65%)
Generalised rhinoorbitocerebral		2 (1.6%)	3 (2.42%)	5 (4.02%)
Pulmonary		0	0	0
Cutaneous		0	0	0
Renal		0	1 (0.81%)	1 (0.81%)
Disseminated	Sinoorbital + pulmonary	1 (0.81%)	1 (0.81%)	2 (1.62%)
	Sinocerebral + pulmonary	0	1 (0.81%)	1 (0.81%)
	Generalised rhinoorbitocerebral + pulmonary	0	1 (0.81%)	1 (0.81%)
	Localised sinus + pulmonary	1 (0.81%)	3 (2.42%)	4 (3.23%)
	Sinoorbital + cutaneous	1 (0.81%)	0	1 (0.81%)
	Localised sinus + cutaneous	0	1 (0.81%)	1 (0.81%)
	Confirmation not available	Patients died within 2 days	2 (1.6%)	0	2 (1.6%)
Recent COVID-19 (persistent RTPCR positive)		10 (8.07%)	0	10 (8.07%)
		26 (20.97%)	98 (79.03%)	124

**Table 12:** Association of site of involvement with the outcome in mucormycosis The chi-square statistic is 23.039. The p-value is 0.00004. The result is significant at p < 0.05.

Site of involvement	Death	Discharge	Total
Cerebral	6 (5,40%)	8 (7.21%)	14 (12.61%)
Localised sinus	1 (0.90%)	61 (54.96%)	62 (55.86%)
Sinoorbital	4 (3.60%)	23 (20.72%)	27 (24.32%)
Disseminated (except those with cerebral involvement and only renal involvement)	3 (2.70%)	5 (4.51%)	8 (7.21%)
Total	14 (12.6%)	97 (87.4%)	111

Mode of ventilation

Out of a total of 124 patients, 67.74% were on room air, 12% on nasal oxygen, 6.4% on NRBM, 5.6% on noninvasive BIPAP, and 8% on invasive mechanical ventilation during the course of their treatment. These modes of ventilation suggest the maximum ventilator support required by the patients during their treatment period. 

Amph-B adverse effects

All our patients received injection lyophilized Amph-B. A significant 44.36% of patients did not have any renal impairment till the end of therapy. Renal impairment was seen in 55.64% of patients, dyselectrolytemia in 56.45%, cardiac side effects (chest pain) in 9.67%, and persistent chills and rigor even after premedication with injection hydrocortisone in 19.35% of patients.

Posaconazole adverse effects

We observed adverse effects of posaconazole such as pedal edema in 7.25%, chills and rigor in 5.64%, dyselectrolytemia in 9.67%, nausea and vomiting in 49.19%.

Surgical treatment

**Figure 3:**
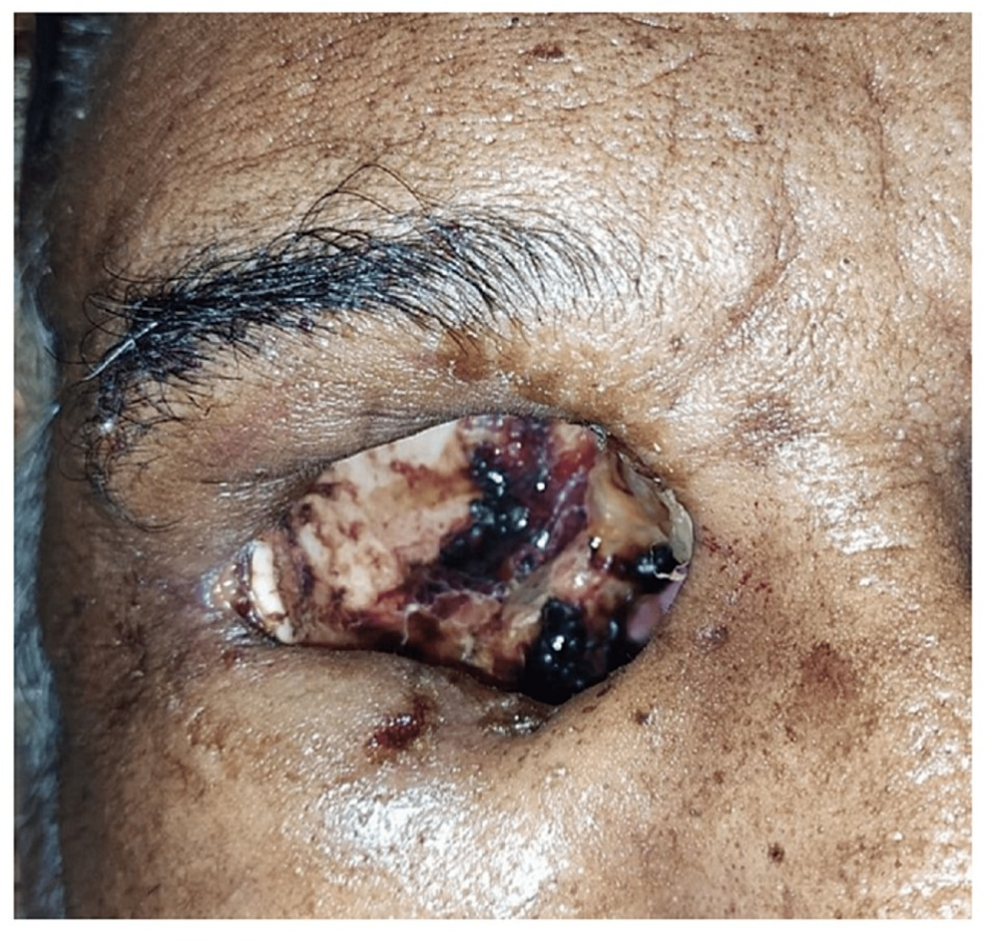
An image of the post-exenteration right eye shows an empty orbital cavity with necrotic tissue and blood clots

As has been described previously, sinoscopy and debridement were done in all patients except those who were persistently COVID-19 positive. A total of 52 patients required radical surgery with the removal of the affected local part. A total of 32 patients required orbital intervention as detailed below (Table 13; Figure 3).

**Table 13:** Surgical interventions

Surgery		Number of patients (out of total 124 patients)
Sinoscopy and debridement		113 (91.13%)
Enucleation/evisceration	Left	12 (9.68%)
	Right	0
Exenteration	Left	4 (3.23%)
	Right	2 (1.61%)
	Bilateral	1 (0.81%)
Orbital decompression	Left	6 (4.84%)
	Right	7 (5.65%)
Partial maxillectomy		8 (6.45%)
Hard palate and tooth removal		12 (9.68%)

Dyselectrolytemia

Mild to severe hypokalemia (76.61%) and hypocalcemia (63.71%) were the most common electrolyte abnormalities observed during the study (Tables 14, 15). We observed QT prolongation in a significant number of patients (72%) after starting injection Amph-B that may be related to electrolyte abnormality (hypocalcemia, hypokalemia, etc.) or direct cardiac toxicity.

**Table 14:** Total number of patients with electrolyte abnormality

Electrolyte	Hyper	Hypo	Normal	Total
Sodium (135-145 mEq/L)	6 (4.84%)	25 (20.16%)	93 (75%)	124
Ionized calcium (1.20-1.40 millimol/L)	3 (2.42%)	79 (63.71%)	42 (33.87%)	124
Magnesium (1.3-2.1 mEq/L)	14 (11.29%)	35 (28.23%)	75 (60.48%)	124

**Table 15:** Total number of patients with an abnormal serum potassium level

Potassium	Hyper (>5.2 mmol/L)	Hypo (subdivided according to severity) (<3.6 mmol/L)			Normal (3.6-5.2 mmol/L)	Total
	5 (4.04%)	Mild (3-3.5 mmol/L)	Moderate (2.5-3 mmol/L)	Severe (<2.5 mmol/L)	24 (19.35%)	124
		22 (17.74%)	34 (27.42%)	39 (31.45%)		
Total number of patients	5 (4.04%)	95 (76.61%)			24 (19.35%)	124

The case fatality rate of MM was 20.97% and the discharge rate was 79.03%.

## Discussion

On an extensive literature search, no large single-centre studies on MM could be found. In one of the meta-analyses done in 2019 by Jeong et al. consisting of 851 patients in the pre-COVID era, diabetes was a major risk factor with 40% of patients, in contrast to our study in which 93.54% of the patients had diabetes [8,9]. In that study, death was reported in 46% (389/851) of the patients. However, in our study, 20.97% of the patients expired. Corticosteroid use alone did not appear to be an independent risk factor in the above-mentioned study. In our study, 52.41% of patients never took steroids in the past six months. Also, in our study, antifungal therapy with early surgical intervention was associated with lower mortality compared to antifungal therapy alone. Similar observations were seen in the above-mentioned meta-analysis also. We also saw 100% mortality in patients in whom surgical therapy was not possible.

A multicentre, retrospective ophthalmological study of 2826 patients conducted in 2021 by Sen et al. showed that rhino-orbito-cerebral mucormycosis (ROCM) was the most common, while in our study, localized sinus infection (50%) was the commonest presentation. Male preponderance (69.35%) was seen in our study as was also observed by Sen et al. There were 87% of patients who had received steroids for COVID-19 in the past and 78% had diabetes, compared to our study where 52% patients had never received steroids and 93.54% had diabetes [10].

A systemic review of 101 worldwide patients done by Singh et al. in 2021 showed that 59.4% of patients presented with an active COVID-19 infection and 40.6% recovered from COVID-19, compared to our study where 20.96% of patients had an active COVID-19 infection, 65.32% had a COVID-19 infection in the past and 13.72% never had COVID. Diabetic ketoacidosis was found in 14.9% of patients, compared to 5.6% in our study. A localized sinus infection was the commonest (88.9%) as in our study (50%) [11].

The case fatality rate ranged from 40% to 52% in the above-mentioned studies compared to 20.97% in our study.

Our study was conducted during an epidemic of MM while the global COVID-19 pandemic was ongoing. Hence, the results of our studies are not representative of those of the general population. Moreover, almost all the patients (93.54%) in our study were diabetics; therefore, a comparison of the MM outcome with a non-diabetic group was not possible.

## Conclusions

We observed that the younger age group (less than 60 years of age) was affected twice as compared to the elderly people. However, the mortality rate was doubled in those who were more than 60 years of age. The male predominance for MM was noted in our study but without any gender predilection for mortality, with an overall case fatality rate of 20.97%. The orbital complaints such as swelling and pain were the most common presenting complaints. We also found that the unhygienic use of mask in diabetic patients during a COVID-19 infection acted as a predisposing factor for MM. Mortality was higher among those patients who had received a higher dose of steroids during COVID infections, implicating a role of steroids in the pathogenesis of MM. The majority of the patients had a past history of a COVID infection, suggesting a complex interplay between COVID and MM pathogenesis, and MM patients with an ongoing COVID-19 infection had a higher mortality. The commonest site of involvement was paranasal sinuses and the best prognosis was seen in those who had localised sinus disease. Those with the cerebral extension of the disease had a poor prognosis. The common side effects of Amph-B were renal function impairment and dyselectrolytemia. Almost half of the patients underwent surgery for the removal of the local part affected by the disease. To conclude, our study derived several factors affecting the outcome of MM, specifically in the context of diabetes and COVID-19.
